# Heritable Changes in Physiological Gas Exchange Traits in Response to Long-Term, Moderate Free-Air Carbon Dioxide Enrichment

**DOI:** 10.3389/fpls.2019.01210

**Published:** 2019-10-14

**Authors:** Aidan David Holohan, Christoph Müller, Jennifer McElwain

**Affiliations:** ^1^School of Biology and Environmental Science, The Earth Institute, O’Brien Centre for Science (E4.47), University College Dublin, Dublin, Ireland; ^2^Institute for Plant Ecology and Interdisciplinary Research Center (IFZ), Justus Liebig University, Giessen, Germany; ^3^School of Biology and Environmental Science, University College Dublin, Dublin, Ireland; ^4^Botany Department, School of Natural Sciences, Trinity College Dublin, Dublin, Ireland

**Keywords:** elevated CO2, acclimation, intrinsic water-use efficiency, FACE, grasslands, leaf gas exchange

## Abstract

Atmospheric carbon dioxide ([CO_2_]) concentrations significantly alter developmental plant traits with potentially far-reaching consequences for ecosystem function and productivity. However, contemporary evolutionary responses among extant plant species that coincide with modern, anthropogenically driven [CO_2_] rise have rarely been demonstrated among field-grown plant populations. Here we present findings from a long-term, free-air carbon dioxide enrichment (FACE) study in a seminatural European grassland ecosystem in which we observe a differential capacity among plant species to acclimate intrinsic water-use efficiencies (WUEs) in response to prolonged multigenerational exposure to elevated [CO_2_] concentrations. In a reciprocal swap trial, using controlled environment growth chambers, we germinated seeds from six of the most dominant plant species at the FACE site [*Arrhenatherum elatius* (L.), *Trisetum flavescens* (L.), *Holcus lanatus* (L.), *Geranium pratense* (L.), *Sanguisorba officinalis* (L.), and *Plantago lanceolata* (L.)]. We found that long-term exposure to elevated [CO_2_] strongly influenced the dynamic control of WUE_i_ in the first filial generations (F_1_) of all species as well as an unequal ability to adapt to changes in the [CO_2_] of the growth environment among those species. Furthermore, despite trait–environment relationships of this nature often being considered evidence for local adaptation in plants, we demonstrate that the ability to increase WUE_i_ does not necessarily translate to an ecological advantage in diverse species mixtures.

## Introduction

Processes that govern guard cell responses to environmental stimuli and the anatomical, morphological, and physiological responses that are driven by both biotic and abiotic pressures have significant implications for interpreting plant–atmosphere interactions (Franks et al., 2012; Franks et al., 2017). Of particular relevance is how plants will adapt gas exchange rates in response to rapidly increasing atmospheric carbon dioxide [CO_2_] concentrations as alterations in this particular atmospheric gas have had profound effects on plant adaptation and evolution in the past (Brodribb et al., 2009; Haworth et al., 2011; McElwain et al., 2009; Leakey and Lau, 2012; Raven et al., 2008).

However, despite the influence of [CO_2_] as a driver of plant evolution historically, there remains little evidence that modern-day plant taxa have or are developing evolutionary responses or adaptations to contemporary [CO_2_] increases (Leakey and Lau, 2012). Yet, growth at elevated [CO_2_] concentrations has often been shown to provoke morphological (McElwain and Chaloner, 1995; Royer, 2001; Woodward and Kelly, 1995) and physiological (Drake et al., 1997; Ainsworth and Long, 2005) stomatal acclimation responses, which limit water loss, maximize carbon acquisition, increase photosynthetic rate, and increase water-use efficiencies (WUEs) through increasing diffusional resistance (Ainsworth and Rogers, 2007).

Acclimation responses to increased [CO_2_], specifically those associated with leaf gas exchange rates, have been demonstrated in numerous studies and are typically the result of long-term exposure to elevated [CO_2_], often resulting in alterations to resource allocation patterns that directly influence leaf photosynthetic and gas exchange rates (Anderson et al., 2001; Chen, 2005; Lee et al., 2011; Rogers and Ellsworth, 2002). Despite the large number of studies that have described acclimation responses to elevated [CO_2_], there are also some studies that demonstrate no such response (Bader et al., 2010; Crous et al., 2011; Herrick and Thomas, 2001; Leakey et al., 2006; Usuda, 2006), and consequently, there may exist a differential acclimation capacity to increasing [CO_2_] among modern plant species.

Notwithstanding the species-specific variation in responses, meta-analytical studies of carbon dioxide enrichment experiments demonstrate a reduction in stomatal conductance rates among modern plant species of approximately 20% when exposed to elevated [CO_2_] concentrations of up to 600 ppm (Ainsworth and Rogers, 2007; Drake et al., 1997; Long et al., 2004; Wullschleger, 1993). However, the lack of stomatal density and/or pore size responses in FACE studies (Ainsworth and Rogers, 2007) argues for a decoupling of morphological and physiological traits over the typical range of [CO_2_] enrichment concentrations. It has been argued that more derived plant groups, specifically angiosperm species that are the focus of most FACE studies, may be uniquely equipped to respond to [CO_2_] enrichment *via* active physiological control as they possess unique mechanisms for detecting and responding to increases in [CO_2_] that are absent from earlier diverging lineages (Brodribb et al., 2009). Opposingly, more recent studies suggest that a diversity of stomatal regulation and environmental sensitivity exists that does not always group simply along phylogenetic lines (Elliott-Kingston et al., 2016; Mcausland et al., 2016). Whatever the mechanisms might be, highly dynamic stomatal control may confer an important WUE_i_ advantage to taxa in terms of their resilience to the increasingly variable climatic conditions predicted in the near future (Hetherington and Woodward, 2003). It may be the case due to the observed differential capacities of individual species to increase WUE that some species will be better suited to future environments than others as increased WUEs will alter the ecological fitness of some taxa in comparison to neighboring competitors (Blumenthal et al., 2013; Grossman and Rice, 2014; Haus et al., 2018; Huxman and Smith, 2001).

Here we test the hypothesis that species endemic to a seminatural grassland community will show an enhanced capacity to improve intrinsic WUE WUE_i_ under elevated [CO_2_] concentrations as a consequence of long-term growth at a marginal FACE CO_2_ enrichment level (480 ppm). We examine if a 17-year enrichment period under FACE conditions (Jäger et al., 2003) led to heritable adaptations of some grassland species rather than all and test if the detected responses persisted when returned to growth under ambient [CO_2_]. We use plant growth chambers to perform a reciprocal swap experiment in which the offspring of plants established under elevated FACE conditions were grown at ambient [CO_2_] concentrations while the offspring (F_1_ generations) of those grown under ambient field conditions were grown under elevated [CO_2_] concentrations. We therefore aim to determine the heritability of particular physiological traits indicating the potential for genetic or epigenetic adaptations. We uniquely assess the potential of modern-day plant evolutionary responses to contemporary [CO_2_] rise.

## Materials and Methods

### FACE Site and CO_2_ Enrichment System

The study site (°32’N and 8°41.3’E at an elevation of 172 m above mean sea level) is located on the outskirts of Leihgestern, close to the city of Giessen in the federal state of Hesse, Germany. Situated on a flood plain of the Lücknbach rivulet, the site covers an area of 4.5 ha. As of 1997, six of the most ecologically similar plots from an original set of 16 previously monitored 100-m^2^ plots were selected as the locations for three ring pairs (three control rings and three CO_2_-enriched rings), with each treatment being assigned to one ring per block at random. CO_2_ enrichment is carried out during daylight hours year-round to +20% above ambient.

Vegetation is classified as an *Arrhenatheretum elatioris* (L.) (Br.- Bl.) *Filipendula ulmaria* (L.) subcommunity (Kammann et al., 2005) and is dominated by the grass species *Arrhenatherum elatius* (L.) and *Holcus lanatus* (L.), with *Sanguisorba officinalis* (L.) and *Plantago lanceolata* (L.) being among the most dominant forbs. The soil is a Fluvic Gleysol with a texture of sandy clay loam over a clay layer at varying depths (FAO classification). A full description of the Giessen FACE site is provided by Jäger et al. (2003).

### Gas Exchange Measurements and Species Selection

Leaf gas exchange measurements were conducted using a CIRAS-2 portable photosynthesis system and PLC (6) cuvette attachment (PP-Systems, Amesbury, MA, USA). A combination of cuvette head plate attachments (4.5 cm^2^, 2.5 cm^2^, and 1.29 cm^2^) was used to maximize the leaf area available for measurements while reducing the amount of uncovered window space in the cuvette head. All gas exchange measurements were taken between 09:00 and 12:00 in the field, and in all cases, conditions in the cuvette head were set to maintain vapor pressure deficit (VPD) below 12 mb (1.2 kPa), leaf temp at 22°C, CO_2_ concentration at either 400 or 480 ppm, and air flow through the cuvette at 200 ml min^-1^.

Initially, photosynthetic irradiance curves (Pn/I) were run to establish the saturating light for photosynthesis. In this case, Pn (photosynthetic rate) was allowed to settle at maximum PAR (photosynthetically active radiation) of 2,000 µmol (photon) m^–2^ s^–1^ before applying a sequence of light settings (1,600; 1,200; 1,000; 800; 600; 400; 200; 100; 50; 0) with an imposed minimum time step of 120 s at each set point. Light-saturated photosynthetic rate (*A*_sat_) was then calculated using the methods of Norman et al. (1991). Pn/I curves were carried out on two plants for every species in every treatment (giving a total of eight light curves per species), and the maximum *A*_sat_ value recorded for each species was then used as the set PAR value for all other gas exchange measurements. In application of the above cuvette conditions, both *A*_sat_ and stomatal conductance (*g_s[opp]_*) were recorded as spot measurements under optimal conditions from three plants per species in all treatments, with measurements taken from at least three leaves per plant. For each species, recordings where taken after both *A*_sat_ and *g_s[opp]_* had reached steady state under cuvette conditions (approximately 30 min).

Changes in WUE_i_ values were measured by imposing a series of stepped increases in [CO_2_] concentrations. Leaves clamped by the cuvette were allowed to settle until stomatal conductance rates stabilized at 400 ppm CO_2_, and once measurements were recorded at this initial concentration, a series of step changes was imposed (200; 400; 750; 1,000; 2,000 ppm). At each step, *A*_sat_ and *g_s[opp]_* were allowed to reach steady state before physiological measurements were recorded. WUE_i_ was then calculated as the ratio of *A_sat/_g_s[opp]_* (Von Caemmerer, 2000). All measurements were carried out on the youngest, fully expanded leaves of herbaceous forbs and the flag leaf of grass tillers.

Species examined in this study include *Arrhenatherum elatius* (L.), *Trisetum flavescens* (L.), *Holcus lanatus* (L.), *Geranium pratense* (L.), *Sanguisorba officinalis* (L.), and *Plantago lanceolata* (L.), all of which are among the most dominant grass and herb species found at the Giessen FACE site.

### Laboratory Processing and Stomatal Morphological Measurements

Plant specimens were randomly harvested from the six FACE rings on the May 21, 2010. Once harvested, plants were dried and pressed before being stored in paper envelopes in a fume hood (extraction rate of 450 m^3^/h) at room temperature until samples could be processed. Maximum stomatal conductance (*g_max_*) measurements are derived from n = 5 stomatal density counts, n = 5 counts of stomatal pore length, and n = 5 counts of guard cell width of the abaxial leaf surface, in the case of hypo-stomatus species, and the summed values of both leaf sides in the case of amphi-stomatus species.

Calculation of theoretical *g_max_* was carried out according to the protocol of McElwain, Yiotis, and Lawson (2016). For all species, the following formula was applied to stomatal data recorded on the adaxial and/or abaxial leaf surface:

(1) $$g_{\max}=\frac{\frac{dw}{v}\;.\;SD\;.\;pa_{\max}}{pd+\frac{\pi }{2}\sqrt{\frac{pa_{\max}}{\pi }}}$$

Where dw = diffusivity of water vapor at 25°C (0.0000249 m^2^ s^-1^), *v* = molar volume of air (0.0224 m^3^ mol^-1^), SD = stomatal density (m^-2^). As it was not possible to determine the precise pore depth for the species assessed in this study, pore depth is considered equivalent to half the width of an inflated, fully turgid guard cell (Franks and Beerling, 2009a, 2009b).

Leaf morphological measurements (stomatal density, stomatal pore length, and guard cell width) where assessed for each leaf from the exact position used for leaf physiological measurements using either clear nail varnish impressions or epifluorescent microscopy. In the case of epifluorescent microscopy, five photomicrographs were recorded at x200 magnification using a Leica (DMLB, Wetzlar, Germany) epifluorescent microscope and auto‐Montage (v.5.03). The SD was estimated by placing a 0.09-mm^2^ grid on the image for each photomicrograph using AcQuis (v.4.0.1.10; Syncroscopy Ltd., Cambridge, UK). In the case of nail varnish impressions, the same number of photo-micrographs was recorded at x200 magnification as before but under standard light microscopy.

### Growth Chamber Conditions

Seeds for all six species were collected in August 2014. For each species, seeds were harvested at random from a minimum of five maternal plants from each of the three ambient and three elevated FACE rings that had been under continuous treatment for 18 years. To capture as much inherent within-species diversity as possible, seeds were taken from widely spaced maternal plants to account for the potential clustering of interrelated species. Harvested seeds for each of the five species where mixed thoroughly and stored in aluminum foil before being transferred to growth chambers for germination and experimental trials.

Seeds harvested from the six rings (three ambient and three elevated) at the University of Giessen’s FACE site were then germinated and grown in two Conviron BDW-40 (Winnipeg, MB, Canada) walk-in growth chambers at University College Dublin’s Program for Experimental Atmospheres and Climate (PÉAC). In a reciprocal swap experiment, seeds collected from both ambient and elevated rings were grown at 400 ppm (ambient) and 480 (+20%) ppm [CO_2_] to determine whether observations of plants grown under elevated [CO_2_] in the field would persist when returned to growth under ambient conditions.

Environmental conditions within chambers (**Supplementary Table 10**) were programmed to match the mean [CO_2_] concentrations of the Giessen field site and, in so far as possible, the climate data for the Hessen region over the course of July/August 2014 when field measurements were carried out and leaf samples were harvested (**Supplementary Table 11**).

Six plants per species (three each from both ambient and elevated FACE conditions) were allowed to establish in 3-liter pots containing a 3:1 potting mixture of multipurpose potting compost (Scotts Horticulture Ltd., Newbridge Co., Kildare, Ireland) and perlite (William Sinclair Horticulture LTD, Chester, UK) and irrigated manually to field capacity every 48 h over the course of the experimental trial (April–December 2014). Plants were positioned randomly within both chambers and rotated on a weekly basis to ensure uniformity of exposure to chamber conditions.

[CO_2_] concentrations were controlled in each chamber using a WMA-4 infra-red gas analyzer (PP Systems, Amesbury, MA, USA). Chamber conditions were consistently maintained for the duration of the experiment in a simulated diurnal program over a 16/8-h light–dark photoperiod (5.00–6.00 incandescent light only of 0–300 µmol m^-2^ s^-1^; 6.00–9.00 light intensity rises from 300 to 600 µmol m^-2^ s^-1^; 9.00–17.00 midday light intensity of 600 µmol m^-2^ s^-1^; 17.00–20.00 light intensity decreases 600 to 300 µmol m^-2^ s^-1^; 20.00–21.00 incandescent light only of 300–0 µmol m^-2^ s^-1^). Ambient atmospheric O_2 _concentrations were monitored using a PP-systems OP-1 O_2_ sensor, and relative humidity was held constant at 70%.

### Data Analysis

Statistical analysis was performed using R (R Core Team, 2012). A generalized linear model was applied to test the effects of stepped increases in [CO_2_] (200; 400; 750; 1,000; 2,000 ppm) on WUE_i_ values, and the influence of either field population source or growth chamber CO_2_ was then added individually to the model as interactive terms. Improvements to model fit due to the inclusion of interacting factors were assessed using Akaike’s information criterion (AIC) scores and calculated R^2^ values [R^2 =^ 1 - the residual deviance (model deviance)/null deviance].

A generalized linear model was applied to test the effects of stepped increases in [CO_2_] (200; 400; 750; 1,000; 2,000 ppm) on WUE_i_ values, and the influence of either field population source or growth chamber CO_2_ was then added individually to the model as interactive terms. Improvements to model fit due to the inclusion of interacting factors were assessed using AIC scores and calculated R^2^ values [R^2 =^ 1 - the residual deviance (model deviance)/null deviance].

## Results

**Figure 1:**
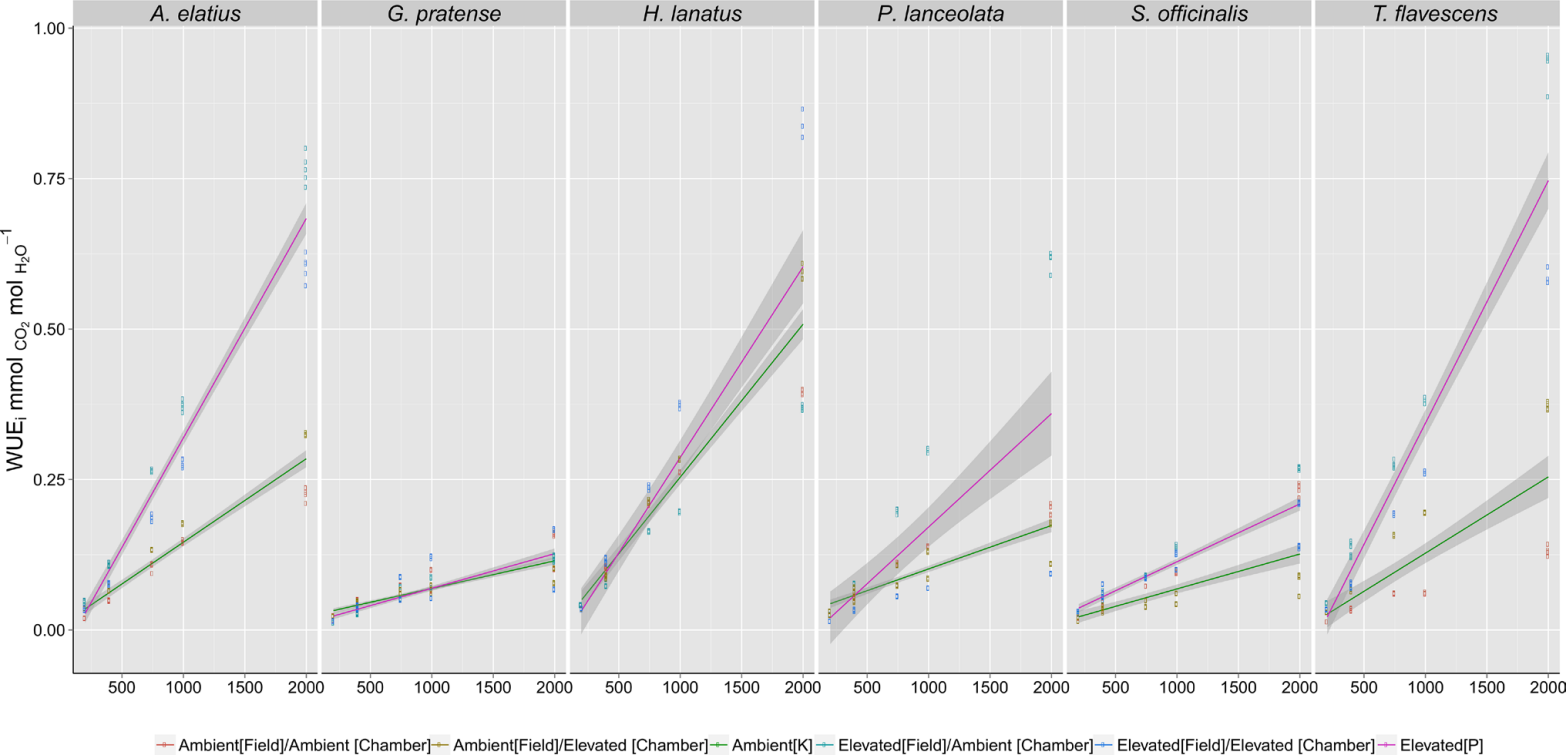
Intrinsicwater-use efficiency (WUE_i_) responses of species grown in growth chambers to step changes in atmospheric carbon dioxide ([CO_2_]) concentrations (200; 400; 750; 1,000; 2,000 ppm). Fitted lines, inclusive of 95% confidence intervals, indicate the differences in response between the F_1_ generations of plants harvested from ambient (Green - 400 ppm) or elevated (Red - 480 ppm) [CO_2_] at the Giessen free-air carbon dioxide enrichment (FACE) site. Significance values (**Table 1**) demonstrate the effect of growth [CO_2_] at both the Giessen FACE site and growth chamber conditions (as interacting terms) on recorded WUE_i_ values. For each species, a minimum of *n* = 5 WUE_i_ values were recorded at each [CO_2_] step (200, 400, 750, etc.), giving a total of *n* = 100 measurements for the F_1_ generation of each individual species.

Long-term exposure to elevated [CO_2_] at the Giessen FACE site directly influenced plant dynamic WUE_i_ responses (response to CO_2_ step changes) when grown in reciprocal swap chamber trials. Stepped increases in [CO_2_] from 200 to 2,000 ppm (**Figure 1**) revealed significant differences (**Table 1**) between plants grown at either ambient (400 ppm) or elevated (480 ppm) [CO_2_] at the Giessen FACE site.

**Table 1:** Generalized linear model outputs predicting the intrinsic water-use efficiency (WUE_i_) response to increasingly elevated atmospheric carbon dioxide ([CO_2_]) concentrations (200; 400; 750; 1,000; 2,000 ppm).

Species	Coefficients	WUE_i_ ∼ CO_2_R x chamber treatment				WUE_i_ ∼ CO_2_R x FACE treatment			
		Estimate	Std. Error	t value	Pr (>|t|)	Estimate	Std. Error	t value	Pr (>|t|)
Arrhenatherum elatius	(Intercept)	-1.03E-02	4.00E-02	-0.258	0.797	-9.88E-02	7.17E-02	-1.378	0.171
	CO_2_R	**2.55E-04**	**2.83E-05**	**9.01**	**4.65E-15**	**3.66E-04**	**9.65E-06**	**37.902**	** <2e-16**
	Elevated chamber treatment	-1.02E-02	5.69E-02	-0.179	0.859				
	CO_2_R x Elevated chamber treatment	-1.28E-05	4.02E-05	-0.318	0.751				
	Ambient FACE treatment					1.16E-01	7.31E-02	1.58	0.117
	Elevated FACE treatment					5.17E-02	7.24E-02	0.715	0.476
	CO_2_R x Elevated FACE treatment					-2.35E-04	1.37E-05	-17.079	<2e-16
		AIC = -77.605, R^2 =^ 0.57, n = 100				AIC = -336.96, R^2 =^ 0.96, n = 100			
Trisetum flavescens	(Intercept)	-4.59E-02	5.55E-02	-0.827	0.41	5.07E-03	2.89E-02	0.175	0.8611
	CO_2_R	**2.88E-04**	**3.91E-05**	**7.351**	**3.00E-11**	**1.23E-04**	**2.04E-05**	**6.059**	**1.74E-08**
	Elevated chamber treatment	2.36E-02	7.85E-02	0.301	0.764				
	CO_2_R x Elevated chamber treatment	-3.74E-05	5.53E-05	-0.675	0.501				
	Elevated FACE treatment					-7.83E-02	4.09E-02	-1.917	0.0577
	CO_2_R x Elevated FACE treatment					**2.91E-04**	**2.88E-05**	**10.097**	**<2e-16**
		AIC = -1.2432, R^2 = ^0.45, n = 100				AIC = -157.84, R^2 = ^0.85, n = 100			
Holcus lanatus	(Intercept)	3.78E-02	1.73E-02	2.185	0.030895	1.45E-02	3.38E-02	0.429	0.669
	CO_2_R	**1.75E-04**	**1.22E-05**	**14.34**	**<2e-16**	**2.42E-04**	**2.38E-05**	**10.175**	**<2e-16**
	Elevated chamber treatment	-9.52E-02	2.45E-02	-3.894	0.000165				
	CO_2_R x Elevated chamber treatment	**2.13E-04**	**1.72E-05**	**12.34**	**<2e-16**				
	Elevated FACE treatment					-4.88E-02	4.78E-02	-1.021	0.3096
	CO_2_R x Elevated FACE treatment					**7.77E-05**	**3.37E-05**	**2.307**	**0.0229**
		AIC = -281.12, R^2^ = 0.93, n = 100				AIC = -120.17, R^2^ = 0.71, n = 100			
Plantago lanceolata	(Intercept)	-6.68E-03	2.53E-02	-0.264	0.792	4.20E-02	2.60E-02	1.614	0.108615
	CO_2_R	**2.09E-04**	**1.78E-05**	**11.726**	**<2e-16**	**6.20E-05**	**1.83E-05**	**3.384**	**0.000918**
	Elevated chamber treatment	4.49E-02	3.26E-02	1.378	0.17				
	CO_2_R x Elevated chamber treatment	**-1.63E-04**	**2.30E-05**	**-7.095**	**5.18E-11**				
	Elevated FACE treatment					-5.42E-02	4.11E-02	-1.319	0.189385
	CO_2_R x Elevated FACE treatment					**1.22E-04**	**2.90E-05**	**4.223**	**4.22E-05**
		AIC = -239.09, R^2 = ^0.63, n = 100				AIC = -169.72, R^2 = ^0.42, n = 100			
Sanguisorba officinalis	(Intercept)	-9.90E-03	9.82E-03	-1.008	0.3146	1.01E-02	9.96E-03	1.018	0.310105
	CO_2_R	**1.30E-04**	**6.92E-06**	**18.796**	**<2e-16**	**5.81E-05**	**7.02E-06**	**8.273**	**3.13E-14**
	Elevated chamber treatment	3.83E-02	1.20E-02	3.186	0.0017				
	CO_2_R x Elevated chamber treatment	**-8.18E-05**	**8.48E-06**	**-9.652**	**<2e-16**				
	Elevated FACE treatment					1.10E-02	1.41E-02	0.782	0.435473
	CO_2_R x Elevated FACE treatment					**3.49E-05**	**9.93E-06**	**3.518**	**0.000554**
		AIC = -628.31, R^2 = ^0.76, n = 100				AIC = -550.19, R^2 = ^0.64, n = 100			
Geranium pratense	(Intercept)	**1.74E-02**	**4.65E-03**	**3.747**	**0.000225**	**2.87E-02**	**4.81E-03**	**5.968**	**8.72E-09**
	CO_2_R	**5.48E-05**	**3.28E-06**	**16.731**	**<2e-16**	**4.14E-05**	**3.39E-06**	**12.207**	**<2e-16**
	Elevated chamber treatment	1.49E-02	6.57E-03	2.259	0.024786				
	CO_2_R x Elevated chamber treatment	-1.81E-05	4.63E-06	-3.909	0.000121				
	Elevated FACE treatment					-7.74E-03	6.80E-03	-1.139	0.256
	CO_2_R x Elevated FACE treatment					**8.73E-06**	**4.80E-06**	**1.82**	**0.07**
		AIC = -1032.4, R^2^ = 0.63, n = 100				AIC = -1015.9, R^2^ = 0.61, n = 100			

Full tables are presented for comparison where either chamber treatment or free-air carbon dioxide enrichment (FACE) treatment is included as interactive terms. Akaike’s information criterion (AIC) and R^2^ values are presented as indicators of model fits, and parameter estimates of model coefficients are presented to evaluate the direction and relative strength of each predictor. Significant predictors for each species are in bold.

Generalized linear models (**Table 1**) demonstrated that elevated [CO_2_] at the FACE site (WUE_i_ ∼ CO_2_R x FACE Treatment) was a significant factor in predicting dynamic WUE_i_ responses for all species (p < 0.05). Chamber treatment (WUE_i_ ∼ CO_2_R x Chamber Treatment) was also a significant factor in the step change responses of *H. lanatus*, *P. lanceolata*, *S. officinalis*, and *G. pratense* (p < 0.05) but not a significant factor for either *A. elatius *or *T. flavescens*. Model fit parameters (AIC and R^2^ values) demonstrated that FACE treatment was a better predictor of dynamic WUE_i_ response than chamber treatment for both *A. elatius *and *T. flavescens,* whereas for all other species, chamber treatment was the more significant factor. Thus, results demonstrate a strong link between the WUE_i_ values of F_1_ generations in growth chambers and the [CO_2_] concentrations parent plants were exposed to at the FACE site. It is also apparent that there is variability among the F_1_ generations of these six species in their ability to acclimate to changes in [CO_2_].

**Figure 2:**
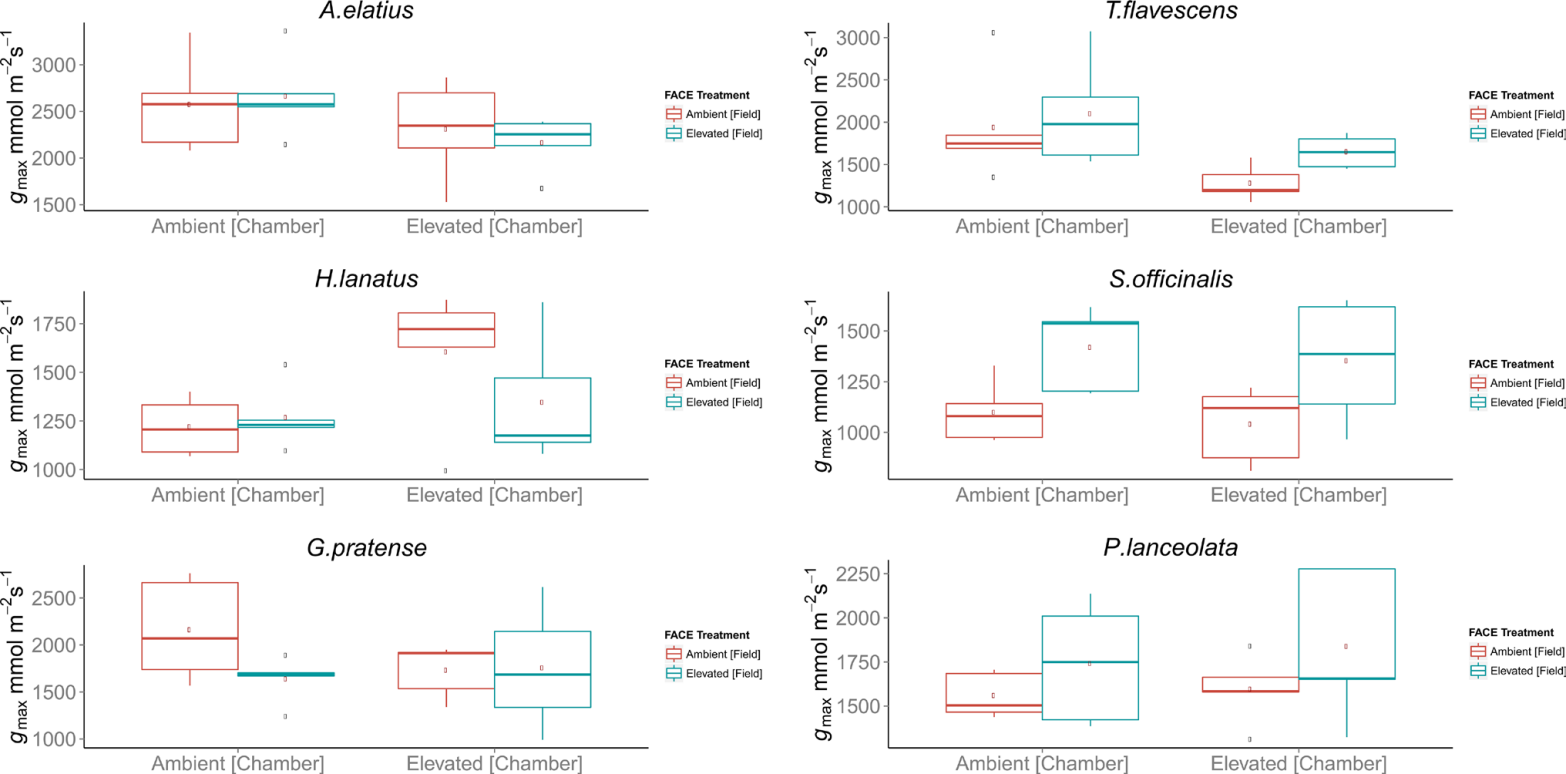
Boxplots of theoretical maximum stomatal conductance (*g_max_*) for the F_1_ generations of field-grown plants harvested from the Giessen free-air carbon dioxide enrichment (FACE) site, which where germinated and grown in environmentally controlled chambers in a reciprocal swap trial. Each chamber treatment (Ambient = 400 ppm, Elevated = 480 ppm) contains the F_1 _generations of plants grown at both ambient (400 ppm) and elevated (480 ppm) atmospheric carbon dioxide ([CO_2_]) concentrations at the Giessen FACE site. Boxplots display the combined mean *g_max_* values of the adaxial and abaxial surface in the case of amphi-stomatus species and abaxial surface only in the case of hypo-stomatus species. In each case, *n* = 5 stomatal density counts, *n* = 5 counts of stomatal pore length, and *n* = 5 counts of guard cell width per leaf surface were used for each species. The top and bottom of the box indicate the upper and lower quartiles, respectively, and the whiskers indicate the minimum and maximum data values. Single data points indicate outliers.

Despite observed physiological responses to moderate [CO_2_] enrichment, chamber trials revealed no significant differences in the *g_max_* values for the F_1_ generations of *H. lanatus*, *A. elatius*,* G. pratense, P. lanceolata*, or *S. officinalis*. The only significant difference in terms of *g_max_* was found for *T. flavescens*. In the case of this species, *g_max_* was significantly reduced for the F_1_ generation of plants grown under ambient [CO_2_] at the FACE site in response to elevated [CO_2_] in growth chambers. In addition, significant differences were found between the F_1_ generations of plants grown under ambient or elevated [CO_2_] at the FACE when grown under elevated [CO_2_] in growth chambers (**Table 2**; **Figure 2**). For a complete breakdown of morphological and physiological measurements see **Supplementary Material (Tables 1 – 10)**.

**Table 2:** Statistical outputs of theoretical maximum stomatal conductance (*g_max_*) responses (of combined abaxial and adaxial surfaces) to chamber treatments ([400 ppm]/[480 ppm]) for the generations of plants grown at either ambient or elevated atmospheric carbon dioxide ([CO_2_]) concentrations in the Giessen free-air carbon dioxide enrichment (FACE) site.

		*g_max_* (TOTAL) mmol m^-2^ s^-1^ (β = 0.5)			
		(400 ppm)		(480 ppm)	
		Ambient (CO_2_)	Elevated (CO_2_)	Ambient (CO_2_)	Elevated (CO_2_)
Arrhenatherum elatius	Mean	1659	1739	1492.2	1398
	Standard deviation	333.74	297.48	348.6	191.38
	Standard error	149.25	133.04	155.9	85.59
	Relative change in mean within chamber (%)	4.6		-6.7	
	Relative change in mean between chambers [Ambient populations (%)]	-11.17812626			
	Relative change in mean between chambers [Elevated populations (%)]	-24.39198856			
	Kruskal-Wallis (within chamber ambient vs elevated populations): chi-squared, P-value	0.5345, 0.4647		0.0982, 0.754	
	Kruskal-Wallis (between chambers ambient populations): chi-squared, P-value	0.0982, 0.754			
	Kruskal-Wallis (between chambers elevated populations): chi-squared, P-value	3.1527, 0.0758			
Trisetum flavescens	Mean	1938	2100	1279	1649
	Standard deviation	653.05	623.73	204.32	84.73
	Standard Error	292.05	278.94	91.37	189.47
	Relative change in mean within chamber (%)	7.7		22.5	
	Relative change in mean between chambers [Ambient populations (%)]	-51.52462862			
	Relative change in mean between chambers [Elevated populations (%)]	-27.32989434			
	Kruskal-Wallis (within chamber ambient vs elevated populations): chi-squared, P-value	0.2727, 0.6015		**4.8109, 0.02828**	
	Kruskal-Wallis (between chambers ambient populations): chi-squared, P-value	**4.8109, 0.02828**			
	Kruskal-Wallis (between chambers elevated populations): chi-squared, P-value	1.8436, 0.1745			
Holcus lanatus	Mean	1220	1268	1605.3	1346
	Standard deviation	145.87	163.83	353.54	324.79
	Standard error	65.23	73.26	158.11	145.25
	Relative change in mean within chamber (%)	3.8		-19.3	
	Relative change in mean between chambers [Ambient populations (%)]	24.00174422			
	Relative change in mean between chambers [Elevated populations (%)]	5.794947994			
	Kruskal-Wallis (within chamber ambient vs elevated populations): chi-squared, P-value	0.5345, 0.4647		0.8836, 0.3472	
	Kruskal-Wallis (between chambers ambient populations): chi-squared, P-value	2.4545, 0.1172			
	Kruskal-Wallis (between chambers elevated populations): chi-squared, P-value	0.0982, 0.754			
Plantago lanceolata	Mean	1559	1741	1595	1837
	Standard deviation	126.51	338.13	190.67	425
	Standard error	56.57	151.21	85.27	190.06
	Relative change in mean within chamber (%)	10.5		13.2	
	Relative change in mean between chambers [Ambient populations (%)]	2.257053292			
	Relative change in mean between chambers [Elevated populations (%)]	5.225911813			
	Kruskal-Wallis (within chamber ambient vs elevated populations): chi-squared, P-value	4.8109, 0.02828		2.4545, 0.1172	
	Kruskal-Wallis (between chambers ambient populations): chi-squared, P-value	0.0982, 0.754			
	Kruskal-Wallis (between chambers elevated populations): chi-squared, P-value	0.0109, 0.9168			
Sanguisorba officinalis	Mean	1098.2	1419	1040.5	1353
	Standard deviation	149.21	204.47	184.94	298.4
	Standard error	66.72	91.44	82.7	133.44
	Relative change in mean within chamber (%)	22.6		23.1	
	Relative change in mean between chambers [Ambient populations (%)]	-5.54541086			
	Relative change in mean between chambers [Elevated populations (%)]	-4.87804878			
	Kruskal-Wallis (within chamber ambient vs elevated populations): chi-squared, P-value	4.8109, 0.02828		2.4545, 0.1172	
	Kruskal-Wallis (between chambers ambient populations): chi-squared, P-value	0.0982, 0.754			
	Kruskal-Wallis (between chambers elevated populations): chi-squared, P-value	0.0109, 0.9168			
Geranium pratense	Mean	2160	1638	1731	1755
	Standard deviation	536.51	240.14	277.54	643.2
	Standard error	239.93	107.39	124.12	287.65
	Relative change in mean within chamber (%)	-31.9		1.4	
	Relative change in mean between chambers [Ambient populations (%)]	-24.78336222			
	Relative change in mean between chambers [Elevated populations (%)]	6.666666667			
	Kruskal-Wallis (within chamber ambient vs elevated populations): chi-squared, P-value	2.4545, 0.1172		0.0109, 0.9168	
	Kruskal-Wallis (between chambers ambient populations): chi-squared, P-value	1.8436, 0.1745			
	Kruskal-Wallis (between chambers elevated populations): chi-squared, P-value	0.0109, 0.9168			

The beta function (β( in this case is an estimation of guard cell depth, which in this case is assumed to be half of the guard cell width (0.5). Significant effects for each species are in bold.

## Discussion

Of the six species included in this study, growth under elevated [CO_2_] at the Giessen FACE site resulted in an enhanced capacity to increase WUE_i_ as [CO_2_] increased from 200 to 2,000 ppm (**Figure 1**). This unique physiological response was persistent among offspring and was nonreversible in at least a single generation. However, four of the six species (*H. lanatus*, *P. lanceolata*, *S. officinalis*, and *G. pratense*) also exhibited significant effects of chamber treatment on the F_1_ generation of field-grown plants in terms of this response, and consequently, we find there exists a differential capacity among these species to adapt to changes in growth environment.

As fluctuations in [CO_2_] concentration directly determine photosynthetic rate and, indirectly, affect plant productivity and fitness, it may be considered to act as a selective pressure driving adaptation and evolution (Bunce, 2018; Watson-lazowski et al., 2016). However, it is not commonly demonstrated in practice, and how it might influence different plant species and/or functional groups is relatively poorly understood, particularly in the context of whole plant communities and naturally fluctuating environments.

A number of previous studies have documented shifts in the relative abundances of species at the Giessen FACE site in response to elevated [CO_2_]. For example, Grüters et al. (2006) demonstrated that both *A. elatius*, and *T. flavescens* have shown relative increases in dominance in the past, whereas *G. pratense*, *P. lanceolata*, and *S. officinalis* have shown a relative decrease.

However, a more recent study by Andresen et al. (2018), examining the effects of elevated [CO_2_] on total aboveground biomass (TAB) at this FACE site, has highlighted a positive response to [CO_2_] enrichment and an overall gain in TAB for the forb species over the full 17-year period. Differences in TAB between species and functional groups (grasses and forbs) were strongly modified by climatic extremes and in particular an unusually late and severe frost episode. This frost event was followed by a reduced abundance of forbs relative to grasses under elevated [CO_2_] for 2 years (2001 and 2002). It is asserted that the damage caused to forb species during a critical phenological stage triggered an initial negative response to elevated [CO_2_], an effect which was not observable for grass species. The implications of the frost event persisted for a further 8 years when forb species began to show a long-term positive response to [CO_2_] enrichment and an overall gain in TAB.

By comparison with the most recent FACE study (Andresen et al., 2018), our findings are contradictory to general assumptions that increasing WUE_i_ should necessarily confer an ecological advantage under future [CO_2_] regimes and, while it does seem to be the case that species preadapted to elevated [CO_2_] show improved WUE_i_ under increasingly elevated [CO_2_], our own study has shown that the ability to adapt to sudden changes in the growth environment is not equal among species. In that sense, the implications of environmental change for diverse plant communities are not clearly understood. Complementary to the Andresen study, an assessment of how a potential [CO_2_] fertilization effect (CFE) at this site might interact with additional climatic variables (temperature, ground water, vapor pressure deficit, etc.) was done. Results demonstrated that increased atmospheric [CO_2_] increases grassland biomass production under average environmental conditions, but the effect is reduced as local conditions become substantially more extreme (wetter, drier, or hotter than average; Obermeier et al., 2016). Here it was suggested that increasing WUE was advantageous in situations where plants may need to cope with additional environmental stressors, such as increased drought stress. However, in situations where water availability is not limiting, the benefit from increased WUE is less obvious. In this study, it was considered that plants adapted to adequate water supply should show improved WUE under elevated [CO_2_] in combination with reduced water availability. This assertion we can confirm with our own results; however, it was also demonstrated that although increases in WUE were greatest in plants exposed to extreme conditions, the CFE in terms of biomass was greater under more moderate environmental conditions where, theoretically, increased WUE is not necessarily an advantage. These results would seem to reinforce our own conclusions and those of Andresen et al. (2018) in that plants may show an enhanced capacity to increase WUE_i _under increasing [CO_2_], all other conditions remaining equal, but where moderate environmental conditions prevail, this adaptation is not particularly useful in terms of increasing TAB.

In terms of morphological traits, we found no significant adaptations in the *g_max_* values for any of the species considered here. As alterations in stomatal morphology can be in terms of size and number (Franks and Beerling, 2009b), the *g_max_* metric was utilized to determine any coordinated morphological response that might ultimately indicate reduced stomatal conductance and additional evidence of a more long-term adaptive response (for a detailed breakdown of stomatal morphological responses, see **Supplementary Material**).

It is possible that these traits are more conserved as they are potentially complexly interlinked with other functional traits. In the case of [CO_2_], responses in terms of stomatal initiation may link with other downstream processes, such as drought-induced hormone signaling (ABA), which in themselves are intrinsically linked [CO_2_] (Chater et al., 2015). By contrast, traits related to stomatal conductance and/or WUEs may be controlled by relatively few genes and consequently respond more rapidly to selection processes (Panio et al., 2013).

A review of plant responses to free-air [CO_2_] enrichment indicates significant capacity for acclimation among modern plant taxa (Anderson et al., 2001; Drake et al., 1997; Maherali et al., 2002) and, in some cases, the capacity for those acclimations to be observable among offspring (Grossman and Rice, 2014; Haus et al., 2018; Li et al., 2019; Nakamura et al., 2011; Saban et al., 2019). However, there are a number of studies that specifically describe a lack of any acclamatory responses (Bader et al., 2010; Crous et al., 2011; Herrick and Thomas, 2001; Leakey et al., 2006; Usuda, 2006). Results presented here may go some way toward explaining this discrepancy as we demonstrate that adaptive responses are not uniform among species (**Figure 1**). Furthermore, there may be poor detection in some cases, and subtle adaptations may go overlooked. This latter point we account for in exposing species to instantaneous increases in [CO_2_] concentrations, as small but definite trait responses may not be readily observable in response to moderate changes in environmental factors but may become apparent under climatic extremes. This is significant as even subtle, almost imperceptible changes in plant gas exchange behavior may strongly influence species responses to extreme weather/climate events in the future.

## Conclusion

The aim of this study has been to determine whether or not any long-term adaptive/acclamatory responses to [CO_2_] enrichment have occurred under FACE conditions since enrichment began at the Giessen FACE site in 1998. Of the six species included in this study, we observed that long-term exposure to elevated [CO_2_] strongly influenced the dynamic control of WUE_i_. This response was unique to plants that had been grown under 480 ppm [CO_2_] at the Giessen FACE site and persisted among the F_1_ generations of those species even when returned to growth at 400 ppm [CO_2_] in growth chambers. We conclude that this particular response was potentially heritable in that it was observable in the F_1_ generation and irreversible despite the imposition of altered growth [CO_2_] concentrations as imposed by reciprocal swap trials.

However, we also observed that plants germinated from seed in growth chambers did not respond to the altered growth conditions uniformly as, despite the influence of FACE conditions, some species were also significantly influenced by chamber treatments. This is a key finding, as despite the evidence for local adaptation in WUE_i_, previous studies of fluctuating TAB at this site demonstrate that an ability to increase WUE_i_ does not necessarily translate to an ecological advantage in diverse species mixtures.

## Author Contributions

AH led the experimental design, carried out the experiment, analyzed the data, and wrote the manuscript. JM and CM were involved in all aspects of the above and provided critical feedback that helped shape the research, analysis, and manuscript.

## Funding

This work was funded by the Earth and Natural Sciences (ENS) Doctoral Studies Programme. The ENS programme is funded by the Higher Education Authority (HEA) through the Programme for Research at Third Level Education, Cycle 5 (PRTLI-5) and is cofunded by the European Regional Development Fund (ERDF). The study was carried out as part of the LOEWE-Excellence cluster FACE_2_FACE.

## Conflict of Interest

The authors declare that the research was conducted in the absence of any commercial or financial relationships that could be construed as a potential conflict of interest.
