# Supplementary material for: Heritable Changes in Physiological Gas Exchange Traits in Response to Long-Term, Moderate Free-Air Carbon Dioxide Enrichment
Source: Front Plant Sci. 2019 Oct 14;10:1210. doi: 10.3389/fpls.2019.01210 (PMC6802601; doi:10.3389/fpls.2019.01210)
Supplement: Supplementary file 1 [file Table_1.docx]

**
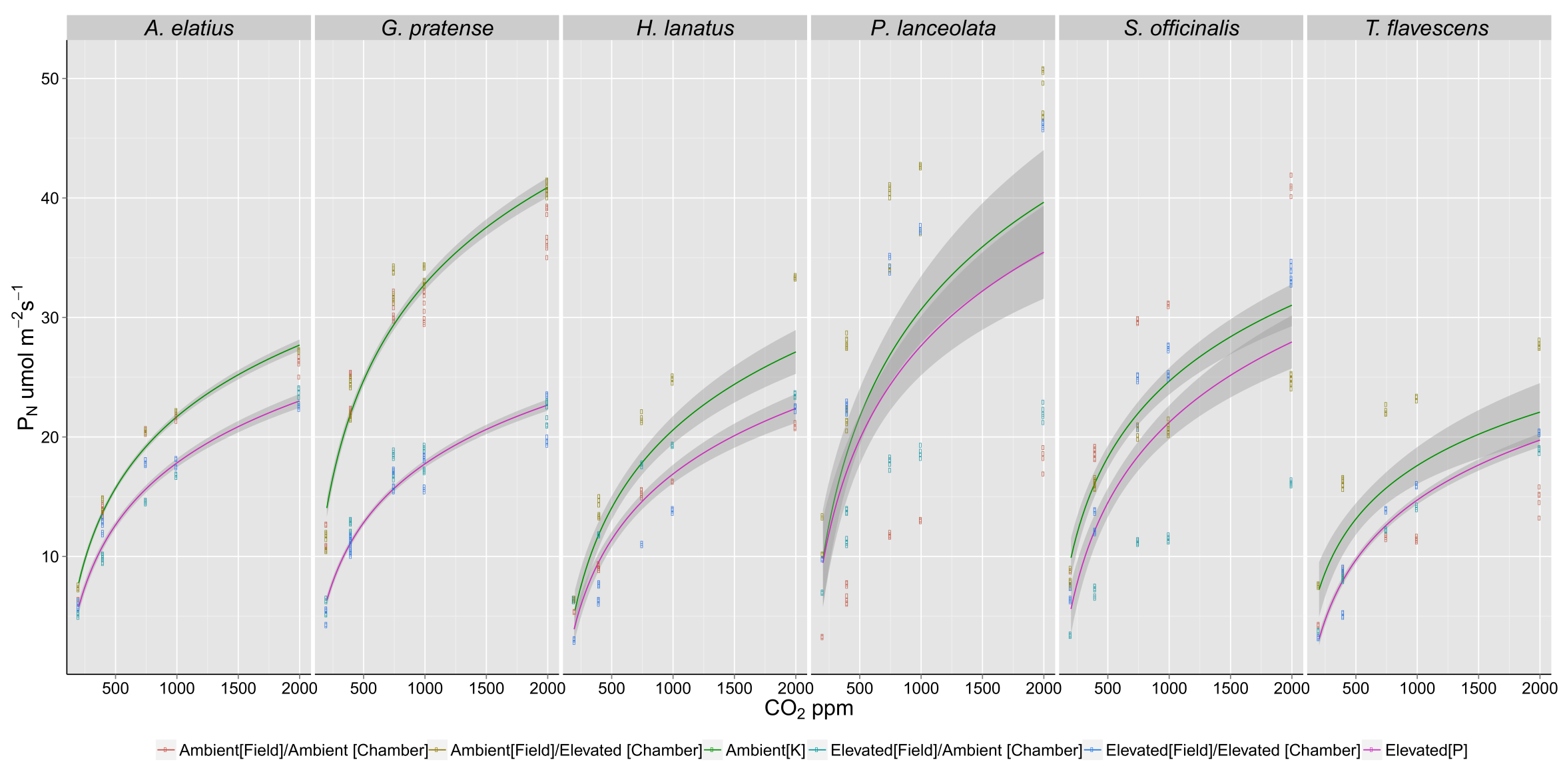
Supplementary figure 1:** Photosynthetic (P_N_) responses of species grown in growth chambers to step changes in [CO_2_] (200, 400, 750 1000 and 2000 ppm) (red dots = Ambient [Chamber], green dots = Elevated [Chamber]). Fitted lines, inclusive of 95% confidence intervals, indicate the differences in response between the F1 generations of plants harvested from ambient (400 ppm) or elevated (480 ppm) [CO_2_] at the Giessen FACE site. Significance values (Supplementary table 1) demonstrate the effect of growth [CO_2_] at both the Giessen FACE site and growth chamber conditions (as interacting terms) on recorded P_N_ values. For each species a minimum of five P_N_ values were recorded at each [CO_2_] step (200, 400, 750, etc.) giving a total of *n* = 50 measurements for the F1 generation of each individual species.

Supplementary table 1: Summary statistics for generalised linear models (GLM’s)

|  | P_N_ ~ CO_2_R | | | | P_N_ ~ CO_2_R x Chamber Treatment | | | | P_N_ ~ CO_2_R x FACE Treatment | | | |
| --- | --- | --- | --- | --- | --- | --- | --- | --- | --- | --- | --- | --- |
| Species | *t-value* | R^2^ | AIC | *p-value* | *t-value* | R^2^ | AIC | *p-value* | *t-value* | R^2^ | *AIC* | *p-value* |
| *A. elatius* | 20.09 | 0.772 | 623.03 | 2.0x10^-16^ | -1.315 | 0.779 | 623.07 | 0.1909 | -0.336 | 0.881 | 548.28 | 0.738 |
| *T. flavescens* | 9.915 | 0.454 | 744.32 | 2.0x10^-16^ | 2.170 | 0.702 | 675.56 | 0.032 | 1.264 | 0.508 | 735.91 | 0.208 |
| *H. lanatus* | 14.39 | 0.637 | 736.12 | 2.0x10^-16^ | 3.570 | 0.702 | 716.31 | 0.001 | -0.824 | 0.699 | 717.31 | 0.412 |
| *G. pratense* | 10.11 | 0.300 | 1756.9 | 2.0x10^-16^ | 0.747 | 0.302 | 1760.1 | 0.456 | -6.285 | 0.910 | 1266.1 | 1.57x10^-9^ |
| *S. officinalis* | 11.39 | 0.422 | 1259.3 | 2.0x10^-16^ | 3.044 | 0.870 | 1052.3 | 0.003 | 0.741 | 0.456 | 1252.2 | 0.4595 |
| *P. lanceolata* | 7.288 | 0.264 | 1220.5 | 1.75x10^-11^ | 7.790 | 0.917 | 896.8 | 1.1x10^-12^ | -0.577 | 0.277 | 1221.8 | 0.565 |

P_N_ ~ CO_2_R describes the model fit in terms of response to step changes in [CO_2_] where CO_2_R is the reference [CO_2_] concentration at each individual step (200, 400, 750, etc.). *t-values*, R^2^, and AIC scores for P_N_ ~ CO_2_R x Chamber Treatment demonstrate the change in model fit when incorporating chamber treatment as the interacting term and P_N_ ~ CO_2_R x FACE Treatment describes any alterations in model fit by including FACE Treatment as the interacting term.
