# Supplementary material for: Heritable Changes in Physiological Gas Exchange Traits in Response to Long-Term, Moderate Free-Air Carbon Dioxide Enrichment
Source: Front Plant Sci. 2019 Oct 14;10:1210. doi: 10.3389/fpls.2019.01210 (PMC6802601; doi:10.3389/fpls.2019.01210)
Supplement: Supplementary file 2 [file Table_2.docx]

**
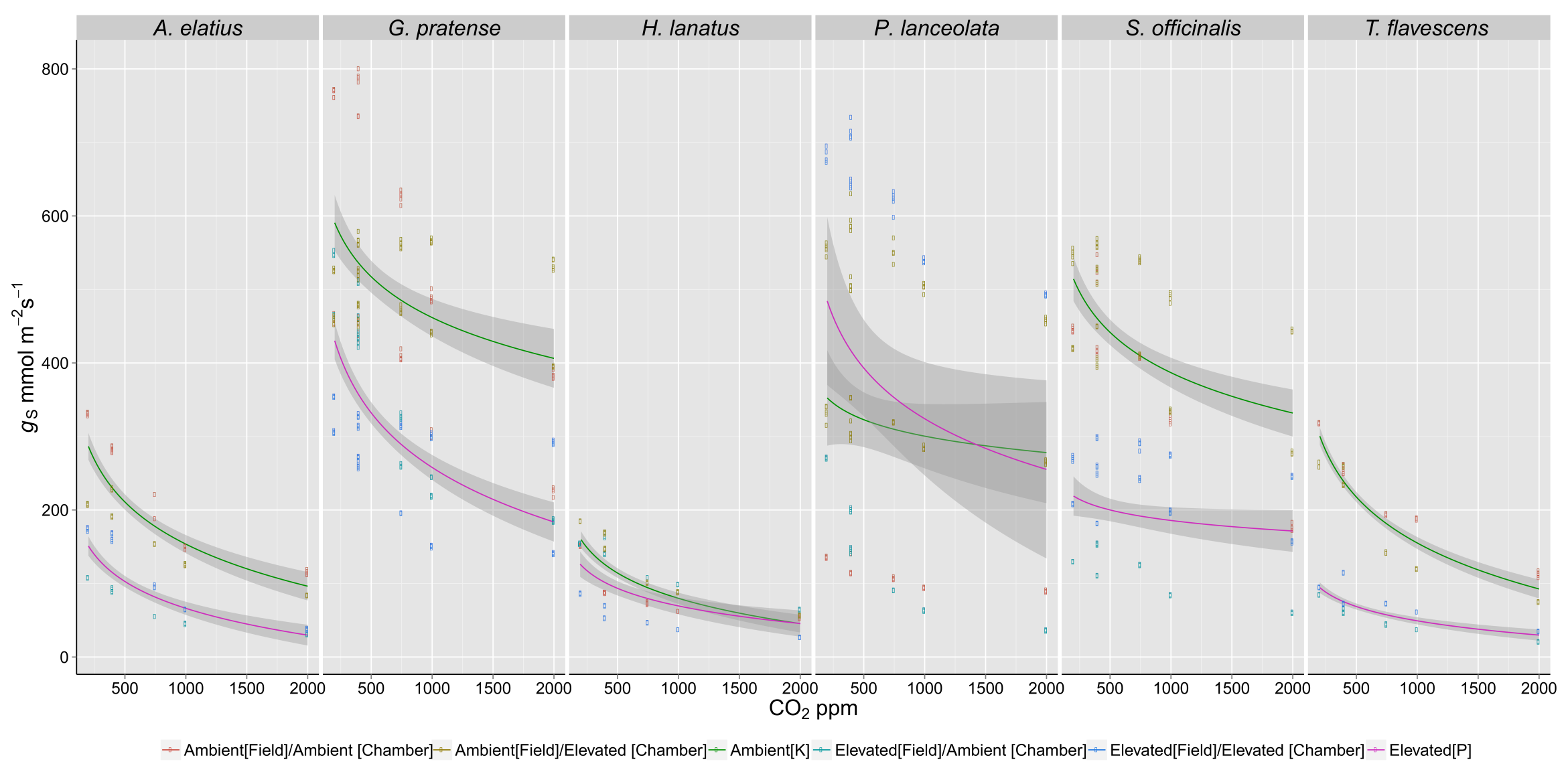
 Supplementary figure 2:** Stomatal Conductance (*g*_s_) responses of species grown in growth chambers to step changes in [CO_2_] (200, 400, 750 1000 and 2000 ppm) (red dots = Ambient [Chamber], green dots = Elevated [Chamber]). Fitted lines, inclusive of 95% confidence intervals, indicate the differences in response between the F1 generations of plants harvested from ambient (400 ppm) or elevated (480 ppm) [CO_2_] at the Giessen FACE site. Significance values (Supplementary table 2) demonstrate the effect of growth [CO_2_] at both the Giessen FACE site and growth chamber conditions (as interacting terms) on recorded *g*_s_ values. For each species a minimum of five *g*_s_ values were recorded at each [CO_2_] step (200, 400, 750, etc.) giving a total of *n* = 50 measurements for the F1 generation of each individual species.

Supplementary table 2: Summary statistics for generalised linear models (GLM’s)

|  | *g*_s_ ~ CO_2_R | | | | *g*_s_ ~ CO_2_R x Chamber Treatment | | | | *g*_s_ ~ CO_2_R x FACE Treatment | | | |
| --- | --- | --- | --- | --- | --- | --- | --- | --- | --- | --- | --- | --- |
| Species | *t-value* | R^2^ | AIC | *p-value* | *t-value* | R^2^ | AIC | *p-value* | *t-value* | R^2^ | *AIC* | *p-value* |
| *A. elatius* | -8.822 | 0.395 | 1343.7 | 1.14x10^-14^ | -0.432 | 0.398 | 1347.1 | 0.666 | 3.827 | 0.815 | 1204.2 | 2.09x10^-4^ |
| *T. flavescens* | -7.053 | 0.296 | 1365.1 | 1.28x10^-10^ | -0.281 | 0.302 | 1368.1 | 0.779 | 12.459 | 0.889 | 1147.4 | 2.34x10^-14^ |
| *G. pratense* | -7.356 | 0.185 | 3107.8 | 3.04 x10^-12^ | 4.821 | 0.261 | 3088 | 2.56 x10^-6^ | 0.480 | 0.622 | 2927.1 | 0.631 |
| *S. officinalis* | -5.029 | 0.124 | 2317.7 | 1.2x10^-6^ | 0.031 | 0.537 | 2264.9 | 0.993 | 3.980 | 0.655 | 2154 | 1.0x10^-4^ |
| *P. lanceolata* | -2.44 | 0.038 | 2062.3 | 0.015 | -0.757 | 0.782 | 1843.4 | 0.450 | -1.043 | 0.0491 | 2064.7 | 0.298 |
|  |  |  |  |  |  |  |  |  |  |  |  |  |

G_s_ ~ CO_2_R describes the model fit in terms of response to step changes in [CO_2_] where CO_2_R is the reference [CO_2_] concentration at each individual step (200, 400, 750, etc.). *t-values*, R^2^, and AIC scores for G_s_ ~ CO_2_R x Chamber Treatment demonstrate the change in model fit when incorporating chamber treatment as the interacting term and G_s_ ~ CO_2_R x FACE Treatment describes any alterations in model fit by including FACE Treatment as the interacting term.
