# Supplementary material for: Heritable Changes in Physiological Gas Exchange Traits in Response to Long-Term, Moderate Free-Air Carbon Dioxide Enrichment
Source: Front Plant Sci. 2019 Oct 14;10:1210. doi: 10.3389/fpls.2019.01210 (PMC6802601; doi:10.3389/fpls.2019.01210)
Supplement: Supplementary file 3 [file Table_3.docx]

Supplementary figure 3: Boxplots of stomatal density for the F_1_ generations of field grown plants harvested from the Giessen FACE site which where germinated and grown in environmentally controlled chambers in a reciprocal swap trial. Each chamber treatment (Ambient = 400 ppm, Elevated = 480 ppm) contains the F_1_ generations of plants grown at both ambient (400 ppm) and elevated (480 ppm) [CO_2_] concentrations at the Giessen FACE site. Boxplots display the mean values of n=9 stomatal density counts for the abaxial leaf surface for each species per treatment. The top and bottom of the box indicate the upper and lower quartiles, respectively, and the whiskers indicate the minimum and maximum data values. Single data points indicate outliers.





Supplementary figure 4: Boxplots of stomatal density for the F_1_ generations of field grown plants harvested from the Giessen FACE site which where germinated and grown in environmentally controlled chambers in a reciprocal swap trial. Each chamber treatment (Ambient = 400 ppm, Elevated = 480 ppm) contains the F_1_ generations of plants grown at both ambient (400 ppm) and elevated (480 ppm) [CO_2_] concentrations at the Giessen FACE site. Boxplots display the mean values of n=9 stomatal density counts for the adaxial leaf surface for each species per treatment. The top and bottom of the box indicate the upper and lower quartiles, respectively, and the whiskers indicate the minimum and maximum data values. Single data points indicate outliers.

Supplementary table 3: Statistical outputs of stomatal density (SD) responses to chamber treatments ([400 ppm]/[480 ppm]) for the F_1_ generations of plants grown at either ambient or elevated [CO_2_] the Giessen FACE site .

|  | | **Stomatal Density (mm^2^)** | | | |
| --- | --- | --- | --- | --- | --- |
|  |  | **[400 ppm]** | | **[480 ppm]** | |
|  |  | Ambient [CO_2_] | Elevated [CO_2_] | Ambient [CO_2_] | Elevated [CO_2_] |
| *Arrhenatherum elatius –*  *Abaxial* | Mean | 66.67 | 41.98 | 54.32 | 50.62 |
|  | Standard Deviation | 29.91 | 13.35 | 8.68 | 8.07 |
|  | Standard Error | 9.97 | 4.45 | 2.89 | 2.69 |
|  | Relative Change In Mean within Chamber (%) | -58.8 | | -7.3 | |
|  | Relative Change In Mean Between Chambers [Ambient Populations (%)] | -22.73564065 | | | |
|  | Relative Change In Mean Between Chambers [Elevated Populations (%)] | 17.06835243 | | | |
|  | Kruskal-Wallis (Within Chamber Ambient Vs Elevated Populations): chi-squared,P-value | 3.4032, 0.06507 | | 0.9158, 0.3386 | |
|  | Kruskal-Wallis (Between Chambers Ambient Populations): chi-squared,P-value | 0.2963, 0.5862 | | | |
|  | Kruskal-Wallis (Between Chambers Elevated Populations): chi-squared,P-value | 2.8138, 0.09346 | | | |
| *Arrhenatherum elatius –*  *Adaxial* | Mean | 82.72 | 83.95 | 72.84 | 82.72 |
|  | Standard Deviation | 47.17 | 14.81 | 26.12 | 13.73 |
|  | Standard Error | 15.72 | 4.93 | 8.71 | 4.57 |
|  | Relative Change In Mean within Chamber (%) | 1.5 | | 11.9 | |
|  | Relative Change In Mean Between Chambers [Ambient Populations (%)] | -13.56397584 | | | |
|  | Relative Change In Mean Between Chambers [Elevated Populations (%)] | -1.486943907 | | | |
|  | Kruskal-Wallis (Within Chamber Ambient Vs Elevated Populations): F,P-value | 0.7155, 0.3976 | | 0.4033, 0.5254 | |
|  | Kruskal-Wallis (Between Chambers Ambient Populations): F,P-value | 1.1357, 0.2866 | | | |
|  | Kruskal-Wallis (Between Chambers Elevated Populations): F,P-value | 0.0529, 0.8181 | | | |

| *Trisetum flavescens –*  *Abaxial* | Mean | 38.27 | 38.27 | 28.4 | 29.63 |
| --- | --- | --- | --- | --- | --- |
|  | Standard Deviation | 5.85 | 8.07 | 8.07 | 5.55 |
|  | Standard Error | 1.95 | 2.69 | 2.69 | 1.85 |
|  | Relative Change In Mean within Chamber (%) | 0.0 | | 4.2 | |
|  | Relative Change In Mean Between Chambers [Ambient Populations (%)] | -34.75352113 | | | |
|  | Relative Change In Mean Between Chambers [Elevated Populations (%)] | -29.1596355 | | | |
|  | Kruskal-Wallis (Within Chamber Ambient Vs Elevated Populations): F,P-value | 0.0395, 0.8424 | | 0.0256, 0.8728 | |
|  | Kruskal-Wallis (Between Chambers Ambient Populations): F,P-value | 6.6572, 0.009876 | | | |
|  | Kruskal-Wallis (Between Chambers Elevated Populations): F,P-value | 5.3138, 0.02116 | | | |
| *Trisetum flavescens-*  *Adaxial* | Mean | 138.3 | 128.4 | 93.83 | 85.19 |
|  | Standard Deviation | 24.28 | 27.27 | 9.79 | 15.71 |
|  | Standard Error | 8.09 | 9.09 | 3.26 | 5.23 |
|  | Relative Change In Mean within Chamber (%) | -7.7 | | -10.1 | |
|  | Relative Change In Mean Between Chambers [Ambient Populations (%)] | -47.3942236 | | | |
|  | Relative Change In Mean Between Chambers [Elevated Populations (%)] | -50.72191572 | | | |
|  | Kruskal-Wallis (Within Chamber Ambient Vs Elevated Populations): F,P-value | 0.5902, 0.4424 | | 1.0295, 0.3103 | |
|  | Kruskal-Wallis (Between Chambers Ambient Populations): F,P-value | 12.2255, 0.0004714 | | | |
|  | Kruskal-Wallis (Between Chambers Elevated Populations): F,P-value | 10.3074, 0.001325 | | | |
| *Holcus lanatus –*  *Abaxial* | Mean | 67.9 | 61.73 | 62.96 | 61.73 |
|  | Standard Deviation | 11.71 | 12.55 | 11.11 | 11.26 |
|  | Standard Error | 3.9 | 4.18 | 3.7 | 3.75 |
|  | Relative Change In Mean within Chamber (%) | -10.0 | | -2.0 | |
|  | Relative Change In Mean Between Chambers [Ambient Populations (%)] | -7.846251588 | | | |
|  | Relative Change In Mean Between Chambers [Elevated Populations (%)] | 0 | | | |
|  | Kruskal-Wallis (Within Chamber Ambient Vs Elevated Populations): F,P-value | 1.483, 0.2233 | | 0.0771, 0.7813 | |
|  | Kruskal-Wallis (Between Chambers Ambient Populations): F,P-value | 1.0295, 0.3103 | | | |
|  | Kruskal-Wallis (Between Chambers Elevated Populations): F,P-value | 0.1059, 0.7449 | | | |

| *Holcus lanatus –*  *Adaxial* | Mean | 58.02 | 61.73 | 64.2 | 60.49 |
| --- | --- | --- | --- | --- | --- |
|  | Standard Deviation | 10.79 | 9.79 | 7.4 | 8.07 |
|  | Standard Error | 3.59 | 3.26 | 2.46 | 2.69 |
|  | Relative Change In Mean within Chamber (%) | 6 | | -6.1 | |
|  | Relative Change In Mean Between Chambers [Ambient Populations (%)] | 9.626168224 | | | |
|  | Relative Change In Mean Between Chambers [Elevated Populations (%)] | -2.049925608 | | | |
|  | Kruskal-Wallis (Within Chamber Ambient Vs Elevated Populations): F,P-value | 0.701, 0.4024 | | 1.3737, 0.2412 | |
|  | Kruskal-Wallis (Between Chambers Ambient Populations): F,P-value | 1.9883, 0.1585 | | | |
|  | Kruskal-Wallis (Between Chambers Elevated Populations): F,P-value | 0.2323, 0.6298 | | | |
| *Plantago lanceolata –*  *Abaxial* | Mean | 133.3 | 156.8 | 151.9 | 161.7 |
|  | Standard Deviation | 16.66 | 29.1 | 32.39 | 28.38 |
|  | Standard Error | 5.55 | 9.7 | 10.79 | 9.46 |
|  | Relative Change In Mean within Chamber (%) | 15.0 | | 6.1 | |
|  | Relative Change In Mean Between Chambers [Ambient Populations (%)] | 12.24489796 | | | |
|  | Relative Change In Mean Between Chambers [Elevated Populations (%)] | 3.03030303 | | | |
|  | Kruskal-Wallis (Within Chamber Ambient Vs Elevated Populations): F,P-value | 3.5636, 0.05906 | | 0.2396, 0.6245 | |
|  | Kruskal-Wallis (Between Chambers Ambient Populations): F,P-value | 1.9027, 0.1678 | | | |
|  | Kruskal-Wallis (Between Chambers Elevated Populations): F,P-value | 0.286, 0.5928 | | | |
| *Plantago lanceolata –*  *Adaxial* | Mean | 138.3 | 156.8 | 149.4 | 161.7 |
|  | Standard Deviation | 19.33 | 16.14 | 28.92 | 17.66 |
|  | Standard Error | 6.44 | 5.38 | 9.64 | 5.88 |
|  | Relative Change In Mean within Chamber (%) | 11.8 | | 7.6 | |
|  | Relative Change In Mean Between Chambers [Ambient Populations (%)] | 7.429718876 | | | |
|  | Relative Change In Mean Between Chambers [Elevated Populations (%)] | 3.03030303 | | | |
|  | Kruskal-Wallis (Within Chamber Ambient Vs Elevated Populations): F,P-value | 4.2565, 0.0391 | | 1.4032, 0.2362 | |
|  | Kruskal-Wallis (Between Chambers Ambient Populations): F,P-value | 0.6518, 0.4195 | | | |
|  | Kruskal-Wallis (Between Chambers Elevated Populations): F,P-value | 0.3575, 0.5499 | | | |

| *Sanguisorba officinalis –*  *Abaxial* | Mean | 176.5 | 211.1 | 145.7 | 184 |
| --- | --- | --- | --- | --- | --- |
|  | Standard Deviation | 23.85 | 38.49 | 19.59 | 11.26 |
|  | Standard Error | 7.95 | 12.83 | 6.53 | 3.75 |
|  | Relative Change In Mean within Chamber (%) | 16.4 | | 20.8 | |
|  | Relative Change In Mean Between Chambers [Ambient Populations (%)] | -21.13932739 | | | |
|  | Relative Change In Mean Between Chambers [Elevated Populations (%)] | -14.72826087 | | | |
|  | Kruskal-Wallis (Within Chamber Ambient Vs Elevated Populations): F,P-value | 4.7739, 0.02889 | | 10.9921, 0.000915 | |
|  | Kruskal-Wallis (Between Chambers Ambient Populations): F,P-value | 6.0146, 0.01419 | | | |
|  | Kruskal-Wallis (Between Chambers Elevated Populations): F,P-value | 3.3321, 0.06794 | | | |
| *Geranium pratense –*  *Abaxial* | Mean | 328.4 | 280.2 | 275.3 | 269.1 |
|  | Standard Deviation | 47.5 | 24.7 | 30.31 | 37.58 |
|  | Standard Error | 15.83 | 8.23 | 10.11 | 12.52 |
|  | Relative Change In Mean within Chamber (%) | -17.2 | | -2.3 | |
|  | Relative Change In Mean Between Chambers [Ambient Populations (%)] | -19.2880494 | | | |
|  | Relative Change In Mean Between Chambers [Elevated Populations (%)] | -4.124860647 | | | |
|  | Kruskal-Wallis (Within Chamber Ambient Vs Elevated Populations): F,P-value | 6.6746, 0.00978 | | 0.0179, 0.8936 | |
|  | Kruskal-Wallis (Between Chambers Ambient Populations): F,P-value | 6.4464, 0.01112 | | | |
|  | Kruskal-Wallis (Between Chambers Elevated Populations): F,P-value | 0.3336, 0.5636 | | | |
