# Supplementary material for: Heritable Changes in Physiological Gas Exchange Traits in Response to Long-Term, Moderate Free-Air Carbon Dioxide Enrichment
Source: Front Plant Sci. 2019 Oct 14;10:1210. doi: 10.3389/fpls.2019.01210 (PMC6802601; doi:10.3389/fpls.2019.01210)
Supplement: Supplementary file 10 [file Table_10.docx]

**Supplementary table 10**: Programed chamber scenarios.

| **Ambient chamber program** | | | | | | |  | **Elevated chamber program** | | | | | | |
| --- | --- | --- | --- | --- | --- | --- | --- | --- | --- | --- | --- | --- | --- | --- |
| Time | Temp | %RH | MHAL | INCAN | O_2_ | CO_2_ |  | Time | Temp | %RH | MHAL | INCAN | O_2_ | CO_2_ |
| 0.00 | 12 | 70 | 0 | 0 | 21% | 400 |  | 0.00 | 12 | 73 | 0 | 0 | 21% | 480 |
| 4.59 | 12 |  | 0 | 0 |  |  |  | 4.59 | 12 |  | 0 | 0 |  |  |
| 5.00 | 12 |  | 0 | 1 |  |  |  | 5.00 | 12 |  | 0 | 1 |  |  |
| 5.20 |  |  |  | 2 |  |  |  | 5.20 |  |  |  | 2 |  |  |
| 5.40 |  |  |  | 3 |  |  |  | 5.40 |  |  |  | 3 |  |  |
| 5.59 |  |  | 0 | 3 |  |  |  | 5.59 |  |  | 0 | 3 |  |  |
| 6.00 | 13 |  | 300 | 3 |  |  |  | 6.00 | 13 |  | 300 | 3 |  |  |
| 9.00 | 18 |  | 600 | 3 |  |  |  | 9.00 | 18 |  | 600 | 3 |  |  |
| 12.00 | 20 |  | 600 |  |  |  |  | 12.00 | 20 |  | 600 |  |  |  |
| 13.40 |  |  |  |  |  |  |  | 13.40 |  |  |  |  |  |  |
| 14.00 | 20 |  | 600 |  |  |  |  | 14.00 | 20 |  | 600 |  |  |  |
| 17.00 | 18 |  | 600 |  |  |  |  | 17.00 | 18 |  | 600 |  |  |  |
| 20.00 | 13 |  | 300 |  |  |  |  | 20.00 | 13 |  | 300 |  |  |  |
| 20.01 |  |  | 0 | 3 |  |  |  | 20.01 |  |  | 0 | 3 |  |  |
| 20.20 |  |  | 0 | 2 |  |  |  | 20.20 |  |  | 0 | 2 |  |  |
| 20.40 |  |  |  | 1 |  |  |  | 20.40 |  |  |  | 1 |  |  |
| 20.59 |  |  |  | 1 |  |  |  | 20.59 |  |  |  | 1 |  |  |
| 21.00 | 12 |  |  | 0 |  |  |  | 21.00 | 12 |  |  | 0 |  |  |
| 23.00 |  |  |  |  |  |  |  | 23.00 |  |  |  |  |  |  |
| 23.59 | 12 | 70 | 0 | 0 | 21% | 400 |  | 23.59 | 12 | 73 | 0 | 0 | 21% | 480 |

**Supplementary table 11**: Programed set points for growth chambers and field meteorological data. Data includes means and standard deviations for peak daytime conditions in the growth chambers and at the Giessen FACE site

|  |  | Ambient CO_2_ concentration | Elevated CO_2_ concentration | Relative Humidity | Max daytime temperature | Max daytime light intensity |
| --- | --- | --- | --- | --- | --- | --- |
|  |  |  |  |  |  |  |
| Ambient Chamber | Set point | 400 ppm | 480 ppm | 70% | 20°C | 600 umol m^-2^ s^-1^ |
|  |  |  |  |  |  |  |
|  | Mean | 404.66 | N/A | 67.02 | 19.87 | 579.36 |
|  | SD | 12.71 | N/A | 4.33 | 0.77 | 65.28 |
|  |  |  |  |  |  |  |
| Elevated Chamber | Set point | N/A | 480 ppm | 70% | 20°C | 600 umol m^-2^ s^-1^ |
|  |  |  |  |  |  |  |
|  | Mean | N/A | 482.66 | 69.99 | 17.57 | 561.97 |
|  | SD | N/A | 3.97 | 2.14 | 2.21 | 114.76 |
|  |  |  |  |  |  |  |
|  | Set point | 400 ppm | 480 ppm | N/A | N/A | N/A |
| Field |  |  |  |  |  |  |
|  | Mean |  |  | 74.01 | 18.95 | 1878.3* |
|  | SD |  |  | 12.63 | 2.92 | 2.63* |

Field data obrtained from federal ministry of transport and digital infrastructure Germany:

http://www.dwd.de/bvbw/appmanager/bvbw/dwdwwwDesktop?_nfpb=true&_pageLabel=_dwdwww_klima_umwelt_klimadaten_deutschland&T82002gsbDocumentPath=Navigation/Oeffentlichkeit/Klima__Umwelt/Klimadaten/kldaten__kostenfrei/kldat__D__gebiete__rasterdaten__node.html?__nnn%3Dtrue
